# The Double-Edged Nature of the Gonadotropin-Releasing Hormone Agonist (GnRHa) Long Protocol: A Case of Paradoxical Ovarian Hyperstimulation During the Expected Downregulation Phase

**DOI:** 10.3390/jcm14144992

**Published:** 2025-07-15

**Authors:** Bernadett Nádasdi, Péter Kovács, Éva Adrienn Csajbók, Károly Wellinger, Anna Vágvölgyi, János Zádori

**Affiliations:** 1 Department of Internal Medicine, Albert Szent-Györgyi Medical School, University of Szeged, 6725 Szeged, Hungary; 2 Dunamenti REK Reproductive Center, Istenhegyi IVF Institute, 1125 Budapest, Hungary; 3 Center of Reproductive Medicine, Department of Obstetrics and Gynecology, Albert Szent-Györgyi Medical School, University of Szeged, 6723 Szeged, Hungary

**Keywords:** gonadotropin-releasing hormone (GnRH), GnRH agonist, GnRH antagonist, ovarian hyperstimulation syndrome (OHSS), pituitary adenoma

## Abstract

**Objectives**: Our aim is to report an uncommon pituitary activation occurring during the desensitization phase of the gonadotropin-releasing hormone agonist (GnRHa) long protocol, a cornerstone of medically assisted reproduction (MAR) therapy, in a young woman. **Results**: We present a case of a 33-year-old female patient with secondary infertility, who exhibited a prolonged and asynchronous follicular development during ovarian stimulation using the GnRH antagonist protocol. Therefore, during a repeat attempt, the long GnRH agonist protocol was employed. Surprisingly, rather than achieving suppression with the agonist, ultrasound detected many large follicles in both ovaries, accompanied by extremely elevated estrogen levels, indicating imminent ovarian hyperstimulation syndrome (OHSS). This unusual phenomenon was also observed during a subsequent attempt using the long protocol in another reproductive center. As part of the work-up to identify the underlying etiology, contrast-enhanced magnetic resonance imaging (MRI) of the sella turcica was performed, which revealed an 11 × 13 × 10 mm pituitary macroadenoma without evidence of pathological hormone secretion. The luteinizing hormone-releasing hormone (LHRH) stimulation test showed a normal luteinizing hormone and follicle-stimulating hormone response. Other abnormalities of the hypothalamo–hypophyseal–target-organ axis were not found. Neurosurgical intervention was deemed unnecessary; radiological follow-up of the lesion was recommended. **Conclusions**: In this case, the clinical presentation was markedly different from the expected suppressive effects of GnRH agonist therapy, with profoundly elevated estrogen levels and clinical signs of imminent OHSS. Notably, hypersensitivity of the adenohypophysis was not demonstrated following a single physiological LHRH stimulation test. However, the presence of a pituitary adenoma identified on MRI raises the possibility that gonadotropin receptor function was altered by the lesion—an effect revealed only after repeated GnRH agonist exposure, resulting in a paradoxical stimulatory response.

## 1. Introduction

The aim of controlled ovarian hyperstimulation during in vitro fertilization (IVF) is to recruit a sufficient number of oocytes for the laboratory procedures [1]. This, in most cases, is achieved using urinary or recombinant gonadotropins [2]. When multiple follicles grow simultaneously, premature luteinization may complicate the treatment and may necessitate cycle cancelation. Gonadotropin-releasing hormone analogs have been introduced into treatment to prevent this. In the 1980s and for nearly 30 years afterward, the gonadotropin-releasing hormone (GnRH) agonist long protocol for ovarian stimulation therapy was considered the gold standard in assisted reproductive procedures [3]. Recently, this treatment has been replaced by GnRH antagonist therapy, the so-called short protocol, due to its shorter treatment duration, lower gonadotropin requirements, and convenience for the patients [4]. In addition, the use of a GnRH antagonist has been associated with a significant reduction in ovarian hyperstimulation syndrome (OHSS) [5]. Meta-analysis and systematic reviews have reported similar clinical efficacy using the GnRH agonist and GnRH antagonist protocols [6]. These protocols are equally likely to be selected primarily among normal responders, but when the treatment outcome is suboptimal using one protocol, the provider typically switches to the other protocol in the subsequent treatment, hoping for improvement both in oocyte quantity and quality. In the case of the luteal long protocol, GnRH agonist administration is started during the mid-luteal phase as either daily s.c. injections or as a depot formulation [2]. Once bound to the pituitary surface GnRH receptors, the agonist initially induces a flare effect and releases the stored luteinizing hormone (LH) and follicle-stimulating hormone (FSH) from the pituitary into the circulation. After 5–7 days of continuous administration, synthetic GnRH agonists, however, induce the downregulation and desensitization of the GnRH receptors and block further FSH and LH release [5,7]. This state of suppression enables the controlled maturation of oocytes without risking premature luteinization. Once the follicles reach maturity, human chorionic gonadotropin (hCG) is administered to induce the final follicular maturation in preparation for retrieval and fertilization [8,9]. OHSS is a serious, potentially life-threatening complication of IVF that is primarily associated with the simultaneous development of too many follicles, but on rare occasions it can be seen in other clinical scenarios [10].

## 2. Patient History

We present the case of a 33-year-old nulliparous female patient. She was first evaluated for primary infertility at age 29. She had an uncomplicated menarche at the age of 13, with regular 26-day menstrual cycles lasting for 5 days. At the initial evaluation, she had a normal body habitus with a body mass index of 18.9 kg/m^2^. She had no clinical signs of hyperandrogenism and had no breast discharge. Physical examination of the thyroid was normal. Transvaginal ultrasound revealed a retroflected uterus with a symmetrical, homogenous myometrium and intact endometrium. Both ovaries appeared to be normal, and the antral follicle count (AFC) was 11. A hysterosalpingogram revealed an intact uterine cavity and bilaterally patent tubes. Her initial hormonal evaluation showed the following results, with reference ranges provided in parentheses. In the early follicular phase (day 3 of the menstrual cycle), thyroid-stimulating hormone (TSH) was measured as 1.34 mIU/L (0.27–4.20); prolactin was measured as 543 mIU/L (102–496); testosterone was measured as 1.31 nmol/L (<2.86); sexual hormone binding globulin (SHBG) was measured as 138.9 nmol/L (26.1–110.0); FSH was measured as 9.6 IU/L (3.5–12.5); LH was measured as 12.0 IU/L (2.4–12.6 IU/L); and estradiol was measured as 166 pmol/L (98–571). In the luteal phase (day 21), progesterone was 43.90 nmol/L (5.82–75.9). At the patient’s first visit to the infertility clinic, as well as at the time of the most recent IVF treatment, the anti-Müllerian hormone (AMH) level was measured, which has remained essentially stable over the years (AMH: 1.76 ng/mL–1.5 ng/mL; reference range: 1.0–4.0 ng/mL).

Her medical history is unremarkable except for the treatment of chronic maxillary sinusitis as an adolescent which was thought to be the cause of persistent headaches. The husband’s andrological examination revealed semen parameters that are suitable for any type of fertility treatment. As the first line of treatment, intrauterine insemination was offered. In preparation for the intrauterine insemination, the patient took 50 mg of clomiphene citrate (Clostilbegyt^®^, Egis Pharmaceuticals Plc., Budapest, Hungary) between day 3–7 of the cycle. Once 1–3 follicles had reached a size of approximately 20 mm, human chorionic gonadotropin (250 mcg recombinant hCG, Ovitrelle^®^, Merck KGaA, Merck Serono, Darmstadt, Germany) was administered to induce ovulation and the insemination followed in 36–40 h. Meanwhile, an endometrial polyp had to be removed hysteroscopically. Due to failed inseminations, in agreement with the patient, we proceeded to IVF. During the course of these IVF treatments, the patient consistently developed an average of 6–10 follicles measuring 14–20 mm in diameter on the day of the ovulation trigger. She received either a 250 mcg recombinant hCG trigger or a dual trigger consisting of hCG in combination with a single dose of 0.2 mg of triptorelin acetate (Gonapeptyl^®^, Ferring Pharmaceuticals, Saint-Prex, Switzerland). The trigger was administered 35–36 h prior to oocyte retrieval. A total of 3, 4, 7, 7, and 9 oocytes were retrieved across different cycles; two additional cycles employing GnRH agonist downregulation were canceled due to the imminent risk of OHSS. Approximately 30% of the retrieved oocytes were mature. In one cycle, fertilization failed entirely, while in two cycles, embryonic development arrested between days 3 and 5. In another cycle, a single blastocyst (Gardner score 2BB) was formed by day 6, cryopreserved, and later transferred during a modified natural frozen embryo transfer (mnFET) cycle. In a separate cycle, two day-three embryos (5 cells, <20% fragmentation, with symmetric blastomeres) were transferred. In the second stimulated IVF cycle, a GnRH antagonist protocol was employed with ovarian stimulation using follitropin alfa (Bemfola^®^, Gedeon Richter Plc., Budapest, Hungary) at 125 IU per day. This resulted in the retrieval of seven oocytes, of which three were mature, three were immature, and one exhibited an abnormal morphology. Semen analysis revealed a concentration of 18 million/mL, 30% progressive motility, and normal morphology in >4% of spermatozoa. Given the patient’s unfavorable reproductive history, intracytoplasmic sperm injection (ICSI) was performed. Three embryos initiated cleavage, and two embryos at the morula stage were transferred on day 4 post-retrieval. Following successful implantation, a monochorionic, diamniotic monozygotic twin pregnancy was diagnosed 26 days post-transfer. Unfortunately, the pregnancy progressed only until 7 weeks and 3 days of gestation, at which point a missed abortion was diagnosed. Three days later, medical termination of the pregnancy was performed. Genetic (karyotype analysis), hematologic, and immunologic causes of the pregnancy loss were excluded. In the meantime, the patient was diagnosed with isolated high diastolic blood pressure, for which methyldopa (Dopegyt^®^, Egis Pharmaceuticals Plc., Hungary) was added to her medication. Apart from this, the patient did not take any other medications on a regular basis.

## 3. Method and Results

**Figure 1:**
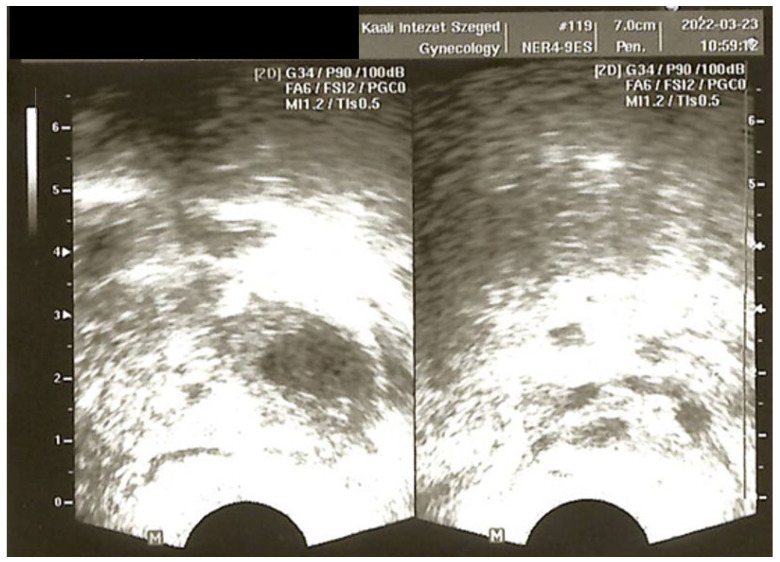
Ultrasound image taken during the first medical examination (before treatment): 2 follicles in the left ovary, including a 12 mm persistent follicle; 4 follicles in the right ovary. (University of Szeged, Institute of Reproductive Medicine.)

**Figure 2:**
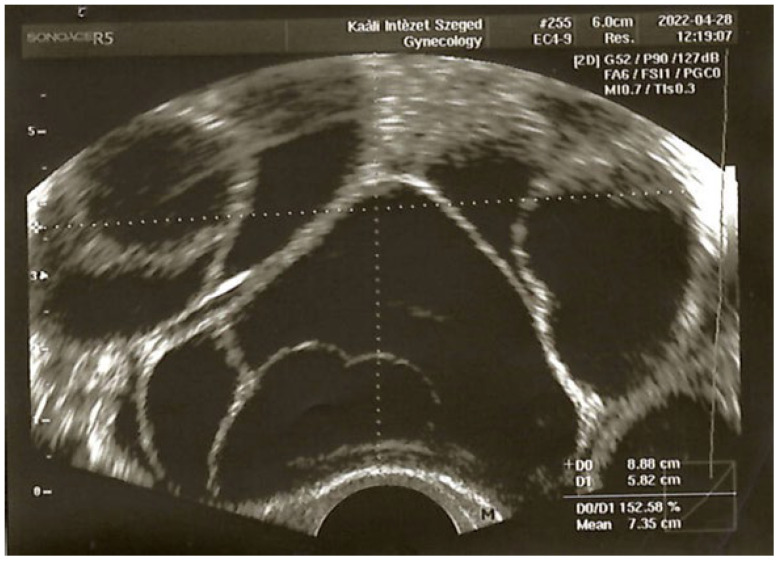
Ultrasound image of a hyperstimulated ovary. Transvaginal ultrasound examination following the desensitization period (10 days) of the GnRH agonist long protocol. Dimensions: 88 × 58 mm. (University of Szeged, Institute of Reproductive Medicine.)

In 2022, the patient had already undergone a total of five IVF treatments, with embryo transfer achieved in only three of them. She became pregnant once during these five treatments, but the pregnancy ended in a missed twin abortion at eight weeks. During a standard GnRH antagonist cycle, delayed oocyte maturation was observed. Therefore, we switched to the long protocol (Figure 1). The GnRH analog buserelin (0.95 mg/day, s.c, Suprefact^®^, Sanofi-Aventis, Paris, France) was initiated on day 20 of the cycle following a negative baseline pelvic ultrasound. Since the expected menstruation (once suppression was reached) did not occur, the patient was brought in for an ultrasound and serum hormone measurement. The ultrasound revealed multiple large follicles, and hormone levels showed no evidence of suppression, but elevated LH (25.9 IU/L), FSH (17.0 IU/L), and extremely high estradiol (>11,010 pmol/L) levels were detected. Due to the absence of menstruation, norethisterone (10 mg/day over 10 days) was administered to induce follicular atresia and menstruation. This paradoxical hyperstimulatory effect—instead of suppression—was unexpected. One potential explanation was that, despite the day 20 start, the initiation of the GnRH agonist fell before ovulation and therefore triggered an initial flare effect and the simultaneous growth of multiple follicles. The findings at the time were consistent with imminent ovarian hyperstimulation (Figure 2).

Subsequently, the patient transferred her care to another fertility clinic. During her first treatment there, she underwent stimulation using letrozole (5 mg/day for 5 days) in combination with recombinant FSH (rFSH) and highly purified human menopausal gonadotropin (hpHMG). Nine oocytes were retrieved, with the majority being immature despite an 18 mm follicle size at trigger and an adequate estradiol level. Eventually, a single blastocyst was cryopreserved on day 6. The frozen embryo was replaced in a modified natural cycle with vaginal progesterone (2 × 200 mg Utrogestan^®^, Besins Healthcare, Laboratories Besins International, Paris, France) luteal support. The frozen embryo treatment did not result in a pregnancy. In order to safely extend the follicular phase to a large follicle size without premature luteinization, the decision was made to try the long protocol. Prior to the initiation of the GnRH agonist (Gonapeptyl^®^ 0.1 mg s.c., Ferring Pharmaceuticals, Saint-Prex, Switzerland), in the late follicular phase, the patient was seen for a vaginal ultrasound that showed a single dominant follicle and an AFC of 9. The GnRH agonist was started on day 20 of the cycle. After 16 days of GnRH agonist administration, there was no sign of menstruation, so the patient was seen for an ultrasound. The scan revealed multiple follicular developments in both ovaries and her serum estradiol level was >11,010 pmol/L. The progesterone level was 13.3 nmol/L and hCG was negative. Gonapeptyl^®^ was discontinued. After one cycle of rest, a repeat attempt was started. During this treatment, to avoid unintended follicular development at the start of the treatment, the GnRH agonist was initially overlapped with oral contraceptives (OCs) (OC use from day 1–14, with GnRH agonist initiated on day 8). When the patient returned for a suppression check after 12 days of Gonapeptyl, the same paradoxical response was observed. Both ovaries were enlarged (6.3 and 5.8 cm) with multiple 18–24 mm follicles, and the serum estradiol level was extremely high at 27,270 pmol/L. At this point, all medications were discontinued, and we waited for follicular atresia while monitoring her using serial ultrasounds and serum hormone measurements. Menstruation occurred within one week, and hormone levels returned to baseline.

**Figure 3:**
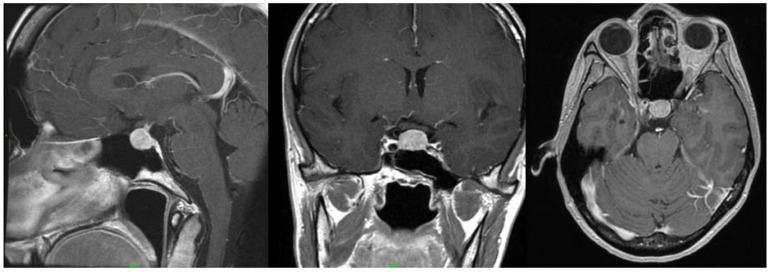
Contrast-enhanced magnetic resonance imaging (MRI) scan of the skull. The pituitary gland is enlarged, measuring 12 × 16 × 13 mm. Most of its tissue is occupied by a structure approximately 11 × 13 × 10 mm in size, which shows more reduced contrast enhancement than the surrounding glandular tissue. This structure exhibits a slight suprasellar extension of 2 mm, slightly elevating the optic chiasm, but without infiltration or compression. (University of Szeged, Department of Radiology).

The adverse response to the GnRH agonist raised a suspicion of underlying pituitary dysfunction. To investigate the cause, contrast-enhanced sella magnetic resonance imaging (MRI) was performed, which revealed an 11 × 13 × 10 mm pituitary macroadenoma (Figure 3). The patient reported no headaches, and a visual field exam revealed no visual field defects. Neurosurgical intervention was not indicated, and annual radiological follow-up was recommended. Hormonal evaluation showed no abnormalities in the hypothalamo–hypophyseal–target-organ axis (Table 1). A luteinizing hormone-releasing hormone (LHRH) stimulation test (Table 2) demonstrated normal LH and FSH responses, which failed to confirm hypersensitivity.

The patient expressed her desire to continue further infertility treatment. Since she had previously shown a normal response to multiple GnRH antagonist cycles, a short protocol was chosen. As of now, at 37 years of age, the patient remains nulliparous despite undergoing seven IVF cycles, three of which did not reach embryo transfer. In several instances, only immature oocytes were retrieved, and embryo development was suboptimal. The failure of oocyte maturation may have a genetic basis, which cannot currently be diagnosed or treated. Oocyte maturation, fertilization, and early embryo development are complex processes under the regulation of numerous genes [11]. Abnormal function of any of these genes could be responsible for the observed suboptimal in vitro functioning and development in our patient. These mutations, however, would not explain her paradox response during GnRH agonist downregulation. Based on her reproductive history, oocyte donation should be strongly considered.

## 4. Discussion

GnRH plays an essential role in female reproduction, regulating oocyte maturation and the menstrual cycle. Its pulsatile release stimulates the anterior pituitary to secrete gonadotropins, LH, and FSH, which in turn drive folliculogenesis and ovulation. One reason for GnRH’s pulsatile secretion is to avoid the downregulation of its receptors in the pituitary [12,13,14]. LH stimulates the aromatase-mediated conversion of androgens to estradiol in granulosa cells in the ovaries and, together with FSH, supports folliculogenesis [15].

The mechanism of GnRH agonist therapy in reproductive medicine—also known as the long protocol—relies on initial stimulation of the pituitary GnRH receptors, leading to a rise in LH and FSH levels. In depot formulations, continuous stimulation causes eventual receptor downregulation and desensitization, suppressing gonadotropin secretion, preventing ovulation, and allowing for follicular synchronization [16]. Due to the reasons discussed in the Introduction, this treatment method was eventually replaced by the so-called short protocol, which involves the use of a GnRH antagonist. Since then, several studies have compared the two therapies, but no clear superiority regarding clinical outcomes has been reported in different patient populations, including poor responders, or patients with endometriosis, adenomyosis, or polycystic ovary syndrome [17,18,19,20,21,22]. Currently, GnRH antagonist therapy is the most commonly used reproductive treatment, and if poor ovarian response is observed, GnRH agonist therapy is considered, as was the case with our patient. We switched to the long protocol after multiple GnRH antagonist cycles in which inadequate folliculogenesis and a poor embryological outcome were reached.

There are no studies in the literature that have examined women with pituitary adenoma undergoing various reproductive procedures. Prolactin-producing pituitary adenomas—which are known as the most frequently occurring adenomas in the hypophysis—impair reproductive function, and in such cases, dopamine agonists are suggested, and surgery is only recommended in cases of macroadenomas that cause visual field loss. The dopamine agonist therapy should only be administered until the desired pregnancy is confirmed [23]. In our patient’s case, there was a non-functioning pituitary adenoma, without compression symptoms, and according to the current guidelines [24] and the findings of the sella MRI, there was no need for surgical therapy. However, there is no data on how the presence of a pituitary adenoma affects the gonadotropin receptors, or how a pituitary adenoma affects hormone production during reproductive treatment.

A limited number of case reports exist in the literature describing a paradoxical ovarian response, including OHSS similar to that observed in our patient during GnRH agonist therapy. In these cases, a GnRH-secreting pituitary adenoma was confirmed. In many of the previously reported cases, relatively elevated baseline LH levels, slightly elevated PRL levels, and spontaneously occurring follicular cysts were observed [25,26,27,28]. In the present case, laboratory investigations did not demonstrate altered baseline hormone levels and the LHRH stimulation tests failed to detect an abnormal response either. However, definitive evidence of hormone production could only be established through the histopathological analysis of the resected pituitary adenoma. According to current guidelines and following neurosurgical consultation, surgical intervention has not yet been undertaken and is not planned.

Gonadotropin secreting adenomas, however, may differ in their initial presentation (in hormone levels, cycle irregularity, spontaneous multi-follicular development, even OHSS). In our case, no abnormalities were detected in the hypothalamic–pituitary hormonal axis, and a normal endocrine response was observed during the physiological LHRH stimulation test. However, following the repeated administration of GnRH agonists—first buserelin and later triptorelin—over a period of 10 days, estradiol levels rose to supraphysiologic levels, accompanied by abdominal bloating, abdominal pain, and enlarged ovaries with several ovarian cysts, which are clinical signs indicating a potential diagnosis of OHSS.

Ovarian hyperstimulation syndrome is a systemic condition that occurs as a complication in approximately 0.5% to 33% of reproductive treatments, although not in every case [29]. In rare instances, OHSS can develop during early spontaneous pregnancy. The modified De Leener classification categorizes spontaneous OHSS into four distinct etiological groups [10]. Among these, the mutation of the FSH receptor, the overproduction of beta-hCG, the hypothyroidism indicated by elevated TSH levels, and the presence of gonadotropin hormones (FSH/LH) secreting pituitary adenomas are recognized as potential causes triggering the OHSS. The pathogenesis of OHSS mainly involves heightened ovarian receptor sensitivity to FSH and/or hCG stimulation. This leads the ovaries to produce disproportionately high levels of steroid hormones, proinflammatory cytokines (such as tumor necrosis factor-alpha, interleukin-1 [IL-1], IL-2, IL-6, and IL-8), and the activation of the renin-angiotensin system. These combined effects increase vascular permeability, causing a pathological rise in interstitial fluid volume and the associated clinical manifestations [30].

The primary clinical symptoms of OHSS include abdominal bloating, abdominal pain, nausea, vomiting, ascites, and generalized swelling. In severe cases, fluid accumulation in the chest cavity (hydrothorax), acute respiratory distress syndrome (ARDS), and pulmonary embolism may occur. These symptoms result from increased vascular permeability throughout the body [31].

Several classification systems and scales exist for the diagnosis and severity assessment of OHSS. The most widely cited is the classification established by the Royal College of Obstetricians and Gynaecologists (RCOG) [32]. According to this classification, the clinical presentation observed in our patient corresponds to threatening, or at most mild OHSS. One possible etiology explaining our case is a gonadotropin-secreting adenoma; however, laboratory results and the completed LHRH endocrine test do not unequivocally support this. The paradoxical response—observed in our case—emerging only after sustained GnRH agonist exposure suggests that the presence of the pituitary adenoma, with or without hormone production, may have altered GnRH receptor function, thereby modifying the downstream gonadotropic response.

## 5. Conclusions

In the case presented, the response to GnRH agonist therapy was entirely contrary to the expected suppressive effect; in fact, markedly elevated estrogen levels and a clinical picture consistent with impending ovarian hyperstimulation were seen. Despite this, hypersensitivity of the adenohypophysis was not demonstrated in response to a single physiological LHRH stimulation test. The presence of a pituitary adenoma, confirmed by MRI, raises the possibility that gonadotropin receptor function was altered by the lesion—an aberration that became clinically evident only after repeated GnRH agonist administration. This rare presentation represents a paradoxical response to a protocol long considered to be the cornerstone of controlled ovarian suppression in medically assisted reproduction.

## 6. Limitations and Future Directions

A potential limitation of this case report is the absence of specific genetic testing. Although multiple gene variants—including candidate OZEMA genes and variants affecting the follicle-stimulating hormone receptor (FSHR) [32,33] and the luteinizing hormone receptor (LHR) [34,35,36]—have been implicated in oocyte maturation failure, we have not yet had the opportunity to perform these analyses. Nonetheless, we recognize the importance of genetic screening in similar cases and are currently exploring options to conduct these investigations in the near future. Further studies may help elucidate the underlying genetic factors contributing to the recurrent oocyte maturation failure observed in this patient.

## 7. What Does This Study Add to Clinical Practice?

This case highlights that in rare instances where ovarian hyperstimulation arises unexpectedly during GnRH agonist therapy—contrary to its intended suppressive effect—a pituitary adenoma should be considered as a potential underlying cause. The presence of such a lesion may alter gonadotropin receptor function, thereby influencing both the physiological response to stimulation and the overall treatment outcome in medically assisted reproduction.

## Figures and Tables

**Table 1:** Hormone profile at the time of the discovery of the pituitary adenoma.

Hormone	Result	Reference Range
Prolactin (mIU/L)	376	102–496
Testosterone (nmol/L)	0.5	<2.86
SHBG (nmol/L)	144	26.1–110.0
FSH (IU/L)	6.6	3.5–12.5
LH (IU/L)	7.5	2.4–12.6
Estradiol (pmol/L)	333	98–571
Progesterone (nmol/L)	66.40	–
DHEAS (µmol/L)	4.09	1.65–9.15
Cortisol (nmol/L)	288	171–536
TSH (mIU/L)	0.80	0.27–4.20
IGF-1 (ng/mL)	149	94.0–252.0
Androstenedione (nmol/L)	2.7	1.0–11.5

DHEAS: dehydroepiandrosterone-sulfate; FSH: follicle-stimulating hormone; IGF-1: insulin-like growth hormone; LH: luteinizing hormone; SHBG: sexual hormone binding globulin; TSH: thyroid-stimulating hormone.

**Table 2:** Result of LHRH test.

Hormone	0 min	30 min	60 min	2 h	4 h
TSH (mIU/L)	0.97	-	0.79	0.64	0.70
PRL (mIU/L)	397	589	492	374	365
Testosterone (nmol/L)	0.82	0.61	0.48	<0.43	0.52
**FSH (IU/L)**	**10.4**	**16.6**	**16.0**	**14.4**	**13.3**
**LH (IU/L)**	**10.9**	**30.2**	**25.4**	**17.6**	**15.6**
E2 (pmol/L)	266	249	205	206	285

E2: estradiol; FSH: follicle-stimulating hormone; LH: luteinizing hormone; PRL: prolactin; TSH: thyroid-stimulating hormone.

## Data Availability

This is a case report. No datasets were generated or analyzed during the current study. Patient data are not publicly available due to privacy and ethical restrictions.
